# Supplementary material for: Determinants of clinical severity in children with sickle cell disease and confirmed asthma
Source: PLoS One. 2026 Apr 28;21(4):e0343757. doi: 10.1371/journal.pone.0343757 (PMC13124003; doi:10.1371/journal.pone.0343757)
Supplement: Attachment — Submitted filename: Response to the Reviewers.PLOS ONE.docx [file pone.0343757.s002.docx]

**Response to the Reviewers**

**Manuscript ID:** PONE-D-26-06629

**Title:** **Determinants of Clinical Severity in Children with Sickle Cell Disease and Confirmed Asthma**

Dear Hilary Genn Givero,

Dear Reviewers,

We sincerely thank the Associate Editor and the two reviewers for their constructive and detailed comments, which have substantially improved the scientific quality and clarity of our manuscript.

## Ethics approval

**Comments to the Author**

## Did you obtain ethics approval for this study?

**Response:** The study was reviewed by the **Ethics Committee of the Centre Hospitalier Andrée-Rosemon (Cayenne, French Guiana)**.
Because the study was conducted as part of routine clinical care in children with sickle cell disease, the committee determined that a formal written approval letter was not required.

The study was conducted in accordance with:

- The Declaration of Helsinki
- French regulations governing research involving human participants
- applicable data protection and confidentiality requirements
- The study complied with the French MR-004 methodology for secondary use of healthcare data

## Retrospective component

Retrospective data were collected from hospital medical records covering the period **between January 1 and December 31, 2025** .Authors accessed **pseudonymized data only**, and had no access to directly identifiable participant information during or after data collection

We hope that these revisions fully address the expectations of the Editor and the reviewers.
We remain at your disposal for any further information.

Yours sincerely,

**Dr Gabriel Bafunyembaka**

On behalf of all authors
