# Supplementary material for: Determinants of clinical severity in children with sickle cell disease and confirmed asthma
Source: PLoS One. 2026 Apr 28;21(4):e0343757. doi: 10.1371/journal.pone.0343757 (PMC13124003; doi:10.1371/journal.pone.0343757)
Supplement: Attachment — Submitted filename: Response to Editorial Comments plos one.docx [file pone.0343757.s003.docx]

Response to Editorial Comments

Manuscript title: Determinants of Clinical Severity in Children with Sickle Cell Disease
Manuscript ID: [PONE-D-26-06629R1]

Dear Editors,

Thank you for your careful review of our submission. We have addressed each of the issues raised as detailed below.

**Comment 1:**
We note that your author list was updated during the revision process. Please complete an Authorship Change Request form.

**Response:**
We have completed the Authorship Change Request form as requested. The change concerns the addition of one author, Ariel Makembi, who was inadvertently omitted from the original submission despite contributing to data collection and manuscript revision. The completed and signed Authorship Change Request form has been uploaded with the submission. All authors have reviewed and approved this change.

**Comment 2:**
We note that there were multiple versions of the manuscript file in your submission.

**Response:**
Thank you for identifying this issue. We have reviewed the submission file inventory and confirm that the most recent version of the manuscript has been retained. No outdated files remain in the submission.

**Comment 3:**
Identifying data were present in the Supporting Information file. Please remove or anonymize personal information and re-upload a fully anonymized dataset.

Response:
We have carefully revised the dataset to ensure full anonymization. All personal identifiers have been removed, including the columns containing date of birth and age. The dataset has been reviewed to ensure that no identifying or potentially identifying information remains. A fully anonymized dataset has been re-uploaded as Supporting Information.

**Comment 4:**
The dataset contained text not in English. Please upload an English-language version.

**Response:**
We have translated all dataset variables and values into English. The revised dataset is now fully provided in English and complies with the journal's data sharing requirements.

We thank the Editorial Team for their valuable guidance and consideration of our manuscript.

Kind regards,

Gabriel Bafunyembaka
Corresponding Author
